# A simple cognitive task intervention to prevent intrusive memories after trauma in patients in the Emergency Department: A randomized controlled trial terminated due to COVID-19

**DOI:** 10.1186/s13104-021-05572-1

**Published:** 2021-05-10

**Authors:** Marie Kanstrup, Laura Singh, Katarina E. Göransson, Beau Gamble, Rod S. Taylor, Lalitha Iyadurai, Michelle L. Moulds, Emily A. Holmes

**Affiliations:** 1 Division of Psychology, Department of Clinical Neuroscience, Karolinska Institutet, 171 77 Stockholm, Sweden; 2 Functional Area Medical Psychology, Karolinska University Hospital, Stockholm, Sweden; 3 Department of Psychology, Uppsala University, Box 1225, 751 42 Uppsala, Sweden; 4 Swedish Collegium for Advanced Study, Uppsala, Sweden; 5 Emergency and Reparative Medicine Theme, Karolinska University Hospital, Stockholm, Sweden; 6 Department of Medicine Solna, Karolinska Institutet, Stockholm, Sweden; 7 MRC/CSO Social and Public Health Sciences Unit & Robertson Centre for Biostatistics, Institute of Health and Well Being, University of Glasgow, Glasgow, UK; 8 Department of Psychiatry, University of Oxford, Oxford, UK; 9 School of Psychology, The University of New South Wales, UNSW Sydney, Australia

**Keywords:** Terminated study, Emergency Department, COVID-19, Intrusive memories, Psychological trauma, Prevention, RCT, Behavioural intervention

## Abstract

**Objective:**

This randomised controlled trial (RCT) aimed to investigate the effects of a simple cognitive task intervention on intrusive memories ("flashbacks") and associated symptoms following a traumatic event. Patients presenting to a Swedish emergency department (ED) soon after a traumatic event were randomly allocated (1:1) to the simple cognitive task intervention (memory cue + mental rotation instructions + computer game "Tetris" for at least 20 min) or control (podcast, similar time). We planned follow-ups at one-week, 1-month, and where possible, 3- and 6-months post-trauma. Anticipated enrolment was *N* = 148.

**Results:**

The RCT was terminated prematurely after recruiting *N* = 16 participants. The COVID-19 pandemic prevented recruitment/testing in the ED because: (i) the study required face-to-face contact between participants, psychology researchers, ED staff, and patients, incurring risk of virus transmission; (ii) the host ED site received COVID-19 patients; and (iii) reduced flow of patients otherwise presenting to the ED in non-pandemic conditions (e.g. after trauma). We report on delivery of study procedures, recruitment, treatment adherence, outcome completion (primary outcome: number of intrusive memories during week 5), attrition, and limitations. The information presented and limitations may enable our group and others to learn from this terminated study.

*Trial registration* ClinicalTrials.gov: NCT04185155 (04-12-2019)

## Introduction

This RCT (ClinicalTrials.gov: NCT04185155) aimed to investigate the effects of a simple cognitive task intervention on intrusive memories ("flashbacks") and other symptoms in patients presenting to a hospital Emergency Department (ED) in Sweden (part of the public healthcare system for all individuals in need of medical emergency care) soon after a traumatic event. Anticipated enrolment was *N* = 148. Primary outcome was the *number of intrusive memories* of the traumatic event (week 5), recorded using a 7-day diary. We predicted that compared to attention placebo, participants receiving the intervention would develop fewer intrusive memories, and less severe related clinical symptoms. We also planned to explore implementation and training aspects in a hospital context. The study was preceded by a pilot RCT (ClinicalTrials.gov: NCT03509792) in the same ED (*N* = 41) [1].

The simple cognitive task intervention targets intrusive memories of trauma (recurrent, distressing memories that spring to mind unbidden). They are a core clinical feature [2, 3] of posttraumatic stress disorder (PTSD) [4]. Targeting intrusive memories is important in its own right to alleviate distress [3, 5], hence the choice of the number of intrusive memories as the primary outcome [6]. Reducing early intrusions might also reduce the risk of developing PTSD [7], and help reduce related clinical symptoms. The intervention comprises a single session with several components: a brief memory reminder cue (to activate the trauma memory), followed by a visuospatial cognitive interference task (playing the computer game ‘Tetris’ on smartphone for at least 20 min following training to use ‘mental rotation’ throughout gameplay [1]). The rationale behind the intervention [8, 9] and more details about the procedure are described elsewhere [1, 5]. Patients are not required to talk about the trauma in detail. The single session (c. 30 min) takes place with a researcher in the ED whilst patients are waiting for medical care. They can engage in self-administered ‘booster’ sessions remotely thereafter. The attention placebo control condition was listening to a podcast (on smartphone) [1, 10] for a similar duration.

Participants were recruited in the ED [11] within c. 6 h of experiencing a traumatic event [6]. As often the first port of call after a traumatic event, the ED provides a setting in which a *preventive* intervention can be evaluated. It enables recruitment of participants with mixed trauma types (e.g., motor vehicle accidents, industrial accidents), and provides an opportunity to evaluate an intervention to trauma-exposed individuals in the *first few hours* post-trauma. Our pilot work indicated that ED patients reported intrusive memories following traumatic events that ranged from minor fall accidents to severe injuries [1].

The current RCT was halted prematurely (10-07-2020) due to the COVID-19 pandemic, after only *N* = 16 participants were randomised. The pandemic prevented recruitment/testing in the ED as it placed significant demands on ED resources and introduced risk of potential infection (due to face-to-face meetings between participants, psychology researchers, ED staff, and patients during study procedures). The host ED was used to receive COVID-19 patients and had a reduced flow of the type of patients who would otherwise present to the ED after trauma in non-pandemic conditions. Many clinical trials internationally have been terminated due to the COVID-19 pandemic [12, 13]**.** Despite termination, we report information about delivery of study procedures (methods), recruitment rates, treatment adherence, outcome completion, attrition, and limitations focusing on pandemic conditions. Our objective is to enable researchers to learn from this experience.

## Main text

### Methods

#### Planned sample size

The planned sample size (*N* = 148) was informed by a pilot RCT (*N* = 41) [1]. Based on the observed between-group difference of *d* = 0.57 for the number of intrusive memories at week 5 (7-day diary), at power of 90% and alpha of 0.05, we would require a sample size of *N* = 65 per group (130 in total). With attrition calculated at 14.2%, we aimed to recruit *N* = 148 in total.^1^

#### Participants

Eligibility criteria included: having experienced or witnessed a traumatic event resulting in admission to the ED, and able to be seen in the ED within approximately 6 h after the traumatic event (day 1); see ClinicalTrials.gov: NCT04185155 for full list.

#### Procedure

Study procedures were carried out by research assistants (RAs) supervised by clinical psychologists (MK/EAH). Session-1 was conducted entirely in the ED (e.g., waiting room/corridors—not separate rooms) on the day patients presented to the ED (day 1). Study procedures were fitted into patients’ time spent in the ED (i.e. whilst waiting for medical care). Potentially eligible patients were identified in collaboration with ED staff, and in consultation with supervisors; patients received further information, and provided written and informed consent. After completing baseline assessments, participants were randomised (parallel assignment, 1:1 ratio) to intervention/control arm using a randomization tool (in electronic data collection platform accessed by RAs away from participants) which used permuted block randomization with random block sizes of 2–10. Participants were not told the condition (intervention/control) to which they were randomized. Researchers in the ED were not blind to condition as they delivered study procedures; however, the outcome assessor was blind. Participants completed their assigned task and received information about how to complete primary and secondary outcomes. Participants in the intervention arm were contacted on day 2 and offered support for self-administered ‘booster’ doses of the intervention as necessary, to target intrusive memories occurring on day 2 onwards. The study was monitored by an independent clinical trial monitoring unit (Karolinska Trial Alliance). We aimed for the study to adhere to CONSORT guidelines.

#### Planned outcomes

Primary outcome was the number of intrusive memories of the traumatic event recorded by participants in a daily diary (morning, afternoon, evening, night) for 7 days during week 5 (5th week after Session-1). This symptom count diary has been used in our previous work [1, 6, 15]. Secondary outcomes included the number of intrusive memories in the daily diary during week 1 (i.e. in the week immediately following Session-1), and symptoms of post-traumatic stress, anxiety and depression (see ClinicalTrials.gov: NCT04185155 for details).

#### Training to deliver intervention/control

Two RAs who delivered the intervention/control task had received training in the pilot study [1]. A third RA only observed and provided administrative support. All RAs received training refreshers before the current trial (e.g., via role-play). Training involved several stages including the RA observing an expert (someone experienced in intervention/control delivery); RA delivering Session-1 procedures to an expert with feedback; real-time/in vivo observation by supervisors of RA with a participant until satisfactory standard; and independent intervention/control delivery by the RA with another RA present. Since RAs had psychology backgrounds (not trained hospital staff), training included guidance on how to work in the ED; e.g., hand hygiene, use of hospital uniform, how to work on a ward without getting in the way [16].

### Results

#### Participant recruitment

**Fig. 1:**
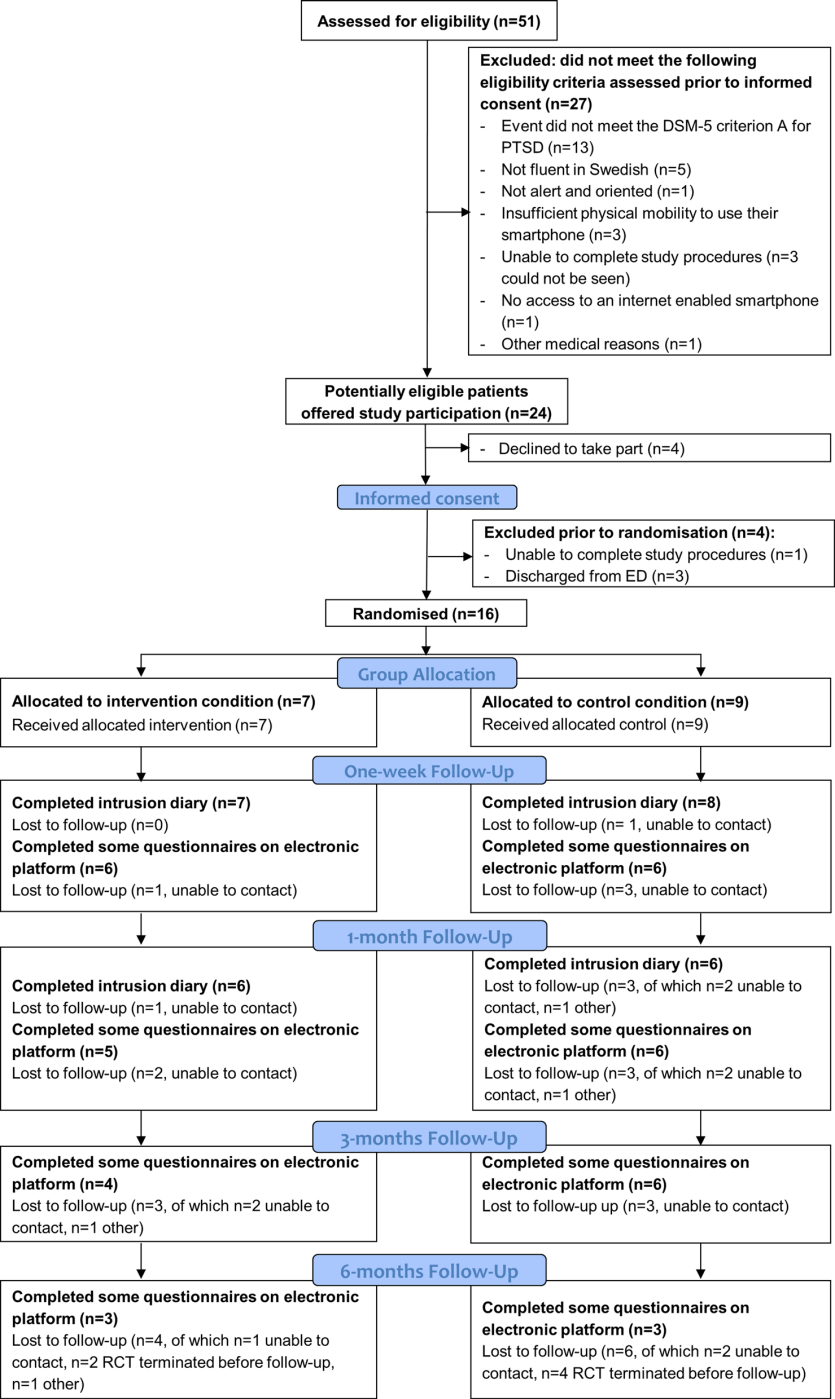
CONSORT participant flow diagram

This RCT was registered on ClinicalTrials.gov on 04-12-2019. Recruitment occurred between 10-12-2019 and 09-03-2020 in the ED (Stockholm, Sweden). Twenty-eight days were spent recruiting in the ED. A supervisor was on site part of the day for 13 of these days for real-time/in vivo supervision, and available for remote supervision otherwise. Of 51 patients approached in the ED and assessed for eligibility, 27 (53%) were excluded for not meeting eligibility criteria assessed prior to informed consent (see Fig. 1: CONSORT participant flow diagram). Of 24 potentially eligible patients offered study participation, 4 (17%) declined to take part, 4 (17%) were excluded prior to randomisation, and 16 (67%) were randomized into the study (Fig. 1). Table 1 shows brief demographics and trauma types.

**Table 1:** Brief demographics and trauma types of full sample.

	Full sample (*N* = 16)
Gender	
Female	*n* = 9
Male	*n* = 7
Other options: Transfemale, Transmale, Genderqueer/non-binary, other identity (option to describe)	*n* = 0
Age	*M* = 40.63 *SD* = 14.45
Type of trauma^a^	
Transportation accident (e.g., car accident, bicycle accident)	*n* = 6
Serious accident at work, home, or during recreational activity (e.g., slip-and-fall injury, threat to limb/extremity)	*n* = 10

^a^The traumatic event leading to ED admission classified using Life Event Checklist 5, LEC-5

#### Treatment adherence

All participants completed their assigned tasks (intervention/control) in the ED in Session-1 (no drop-outs), indicating high acceptability of both arms. Three participants in the intervention condition received remote ‘booster’ sessions via phone to target remaining intrusive memories, which proved possible practically and acceptable to participants.

#### Outcome completion and attrition

Completion rates of the intrusive memory diary were 75% for week 5 (primary outcome), and 94% for week 1 (secondary outcome). Completion of other secondary outcomes (electronic platform) were 75% at one-week, 69% at 1-month, 63% at 3-months and 38% at 6-months (note: the electronic platform was disabled prior to six participants’ 6-months follow-up; of those who received 6-months follow-ups, 60% completed). The main reason for attrition was that participants could not be contacted. No adverse events related to study procedures were reported. No serious adverse events were reported.

#### Trial termination: COVID-19 pandemic prevented recruitment/testing in ED

As the impact of the pandemic became apparent, we ceased recruitment/testing in the ED. We followed developments in the pandemic and as the situation worsened in Sweden, it was clear that continuation of the RCT would not be feasible. We decided to prematurely terminate the study on 10-07-2020. The electronic platform was disabled by the clinical trial monitoring unit on 29-07-2020. The sample (*N* = 16) comprises 11% of anticipated enrolment (*N* = 148). Thus, the terminated study data are insufficient to draw meaningful inferences for between-group comparisons e.g. regarding intervention efficacy. The full RCT was designed to provide 90% power to detect a medium effect size (*d* = 0.57 observed in pilot RCT), and the current sample of *N* = 16 would provide only around 19% power to detect similar effect sizes. Conducting underpowered analyses is not recommended as it (i) lowers the chance of a positive result being true (lowers positive predictive value), (ii) lowers the chance of discovering a true effect, and (iii) inflates the estimate of effect size when a true effect is discovered [17]. We thus conducted no formal analyses on planned group comparisons and data are not reported.

### Discussion

The terminated study indicates feasible recruitment, albeit based on a small sample. Only 17% of patients approached who were potentially eligible and were offered study participation declined. Some other studies in the ED (after trauma) report a higher proportion of eligible patients declining to participate (e.g. 88%-psychotherapy trial, 58%-pharmacological trial [18]). All seven participants randomized to the intervention completed treatment, indicating high acceptability (treatment discontinuation is used to indicate acceptability in [19]); likewise all completed the control condition.

The team had spent months learning about and adapting to working in the ED, and adopting ED standard hygiene/safety procedures under ‘normal’ circumstances, i.e. establishing a sense of embeddedness of the research team in the ED environment and giving researchers the opportunity to understand the ED context, as well as potential obstacles to and facilitators of recruitment and intervention delivery. Integration of psychology research staff into the ED environment in this trial and in our pilot [1] highlight how psychological researchers can successfully work in the ED alongside ED staff in non-pandemic conditions. However, the pandemic brought another level of challenges of being in the ED to which the team could not adapt.

Exposure to traumatic events during the COVID-19 pandemic appeared a rising concern for healthcare staff. Thus, we noted the need for an intervention for healthcare *staff* exposed to traumatic events as part of their work. Working closely with staff within the hospital had allowed us to establish collaborations and gain insights. Wellbeing of healthcare staff became a priority as the pandemic unfolded [20]. Accordingly, following termination of the current trial, we swiftly adapted study procedures for remote delivery and commenced a new trial (ClinTrials.gov: NCT04460014) targeting intrusive trauma memories in healthcare staff.

## Limitations

This RCT was prematurely terminated. Numerous aspects of the ED environment were challenging to navigate during the pandemic, e.g. the uncertain situation, risk regarding virus transmission, and increased need for personal protective equipment. Reliance on face-to-face procedures, alongside reduced flow of patients presenting to the ED after a traumatic event, meant we were unable to continue this study.

The study described to participants during the informed consent process was significantly larger in scale than the final terminated trial reported here. The planned trial—with a much larger sample—would have afforded greater protection of participants’ privacy, particularly given the unique ED site. Owing to such considerations, the current data has not been made publicly available. Overall, lessons learned include the need for post-trauma interventions that are suitable for participants under pandemic conditions [20] i.e. remote recruitment/delivery.

## Data Availability

Data related to delivery of study procedures, recruitment, treatment adherence, outcome completion and attrition are reported within this article. Individual data for these 16 participants are not publicly available owing to considerations regarding the protection of participants’ privacy. Study materials may be made available upon reasonable request with an appropriate materials transfer agreement (MTA) with Uppsala University.

## Abbreviations

CTR: Clinical Trials Registration
ED: Emergency Department
PTSD: Posttraumatic stress disorder
RA: Research Assistant
RCT: Randomised Controlled Trial

## Footnotes

1 There is a discrepancy of *N* = 2 between the *N* = 148 in the CTR (NCT04185155) and the *N* = 146 reported in (1), due to calculations being based on either *N* = 42 participants randomized or *N* = 41 final sample for analysis. N = 146 is based on the corrected attrition rate of 12.2% detected during the review process for (1).
